# ISN Forefronts Symposium 2015: The Evolution of Hypertension–Old Genes, New Concepts

**DOI:** 10.1016/j.ekir.2016.08.003

**Published:** 2016-08-06

**Authors:** Morag K. Mansley, Jessica R. Ivy, Matthew A. Bailey

**Affiliations:** 1 The British Heart Foundation Centre for Cardiovascular Science, The University of Edinburgh, Edinburgh, UK

**Keywords:** blood pressure, evolution, hypertension, inflammation, kidney, pressure natriuresis, salt

## Abstract

Hypertension is known as the “silent killer,” driving the global public health burden of cardiovascular and renal disease. Blood pressure homeostasis is intimately associated with sodium balance and the distribution of sodium between fluid compartments and within tissues. On a population level, most societies consume 10 times more salt that the 0.5 g required by physiological need. This high salt intake is strongly linked to hypertension and to the World Health Organization targeting a ∼30% relative reduction in mean population salt intake to arrest the global mortality due to cardiovascular disease. But how does a habitually high-salt diet cause blood pressure to rise? In this focused review, we discuss 2 “evolutionary medicine” concepts, presented at the ISN Forefront Meeting “Immunomodulation of Cardio-renal Function.” We first examine how ancestral variants in genes that conferred a selection advantage during early human development are now maladaptive. We then discuss the conservation of “renal” sodium transport processes across multiple organ systems, including the brain. These systems influence sodium appetite and can exert an often-overlooked effect on long-term blood pressure control.

Hypertension is known as the “silent killer”: it rarely causes symptoms and is the chief modifiable risk factor driving a global health crisis of cardiovascular and renal disease.^1^ Hypertension exerts a major socioeconomic burden, costing in the United States, for example, more than 46 billion dollars per year to manage in terms of direct health care and associated costs.^2^ At the millennium it was estimated that 972 million adults had hypertension; a 60% increase is predicted by 2025, which means that approximately 1.5 billion adults will have hypertension within the next decade.^3^ To arrest this increase in prevalence, the World Health Organization (WHO) proposes a dual strategy of improving access to inexpensive and effective therapeutic agents alongside education to improve long-term cardiovascular health through lifestyle changes. A key target here is an approximately 30% relative reduction in mean population salt intake. In most countries the average salt (NaCl) intake is 9 to 12 g per person per day, against the WHO recommended intake of 5 g per day.^4^ Although the relationship between salt intake and cardiovascular mortality is u-shaped, targeting salt reduction toward the recommended daily allowance (RDA) would be beneficial^5^: based on the INTERSALT study, long-term compliance would lower blood pressure and significantly reduce cardiovascular events later in life.^6^

### How Does Salt Increase Blood Pressure?

What is the relationship between salt intake that is habitually high and long-term blood pressure? This is difficult to gauge, as most clinical studies estimate salt intake from measurement of 24-hour urinary sodium excretion and recent research clearly shows that this is not an effective index of intake.^7^ Nonetheless, hypertension research has been strongly influenced by the computational modeling of Guyton *et al.*, which placed renal function at the center of long-term blood pressure homeostasis.^8^ The control of effective intravascular volume through urinary excretion of salt and water offsets any perturbation, stabilizing blood pressure around the individual’s set point. For example, an increase in arterial pressure will increase renal arterial pressure and, in turn, will cause blood flow through the medullary vasa recta to rise. This hemodynamic effect promotes the release of a variety of paracrine factors, such as adenosine triphosphate (ATP) and nitric oxide, which directly inhibits sodium transport in the proximal tubule, thick limb of Henle, and distal nephron. This vascular−tubular cross-talk underpins the pressure natriuresis response, that is, the direct relationship between sodium excretion and renal perfusion pressure.^9^ If pressure natriuresis achieves long-term stability of blood pressure, then it follows that hypertension can be sustained only if the renal response to elevated blood pressure is impaired: that is, hypertension is caused by renal dysfunction.^10^ Indeed, it is well documented that the acute pressure natriuresis curve is right-shifted in hypertension; more importantly, the gradient is often blunted. How such acute “loss of function” integrates into chronic blood pressure control is not very well defined. It may initially manifest as loss of the normal nocturnal dip, with blood pressure remaining high to facilitate sodium excretion and maintain balance.^11^ It is also argued that the acute pressure natriuresis mechanism is not the only—or indeed, not the most important—mechanism of sodium and fluid homeostasis^12^: a large body of work in humans, recently reviewed,^13^ finds that high salt intake can increase blood pressure without inducing volume expansion. We also find this in murine models of glucocorticoid hypertension.^14^, ^15^ In both situations, hypertension seems to reflect increased vascular tone and/or enhanced activity of the sympathetic nervous system rather than sodium retention and volume expansion.

It is certain that chronically elevated blood pressure reflects interactions between multiple systems (Figure 1), but this article will not discuss the relative merits of renocentric, vasculocentric, or neurogenic views of hypertension; rather, we focus on the evolution of hypertension, discussing emerging concepts presented at the ISN Forefront Meeting “Immunomodulation of Cardio-renal Function,” held in Shenzhen, China. First, we examine the hypothesis that the hypertension pandemic reflects a discord between our ancestral genes and our current high-salt environment. What can “Evolutionary Medicine” tell us about high blood pressure? Second, the molecular pathways controlling salt reabsorption in the kidney are expressed in other areas important for salt balance, including the brain. We discuss recent research showing that these central pathways can influence salt intake and blood pressure without altering renal function.

**Figure 1:**
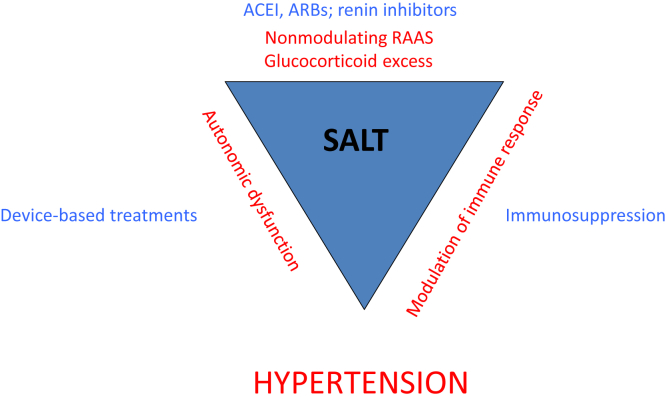
The hypertensive storm. There is strong evidence for a dynamic interaction among hormonal (chiefly the renin−angiotensin−aldosterone system), immune, and autonomic nervous systems in the physiological regulation of blood pressure. In the context of a high-salt diet, these interactions may become maladaptive, causing hypertension. Antagonists of the RAAS are front-line antihypertensive treatments, and device-based interventions are targeting the nervous system. The World Health Organization advocates reducing dietary sodium intake to ∼5 g/d. ACEIs, angiotensin-converting enzyme inibitors; ARBs, angiotensin receptor blockers; RAAS, renin−angiotensin−aldosterone system.

### Ancestral Genes and the Evolution of Hypertension

As recently reviewed,^16^ the processes through which sodium balance is regulated have evolved over millennia. The renin−angiotensin system, for example, emerged approximately 400 million years ago in the Paleozoic era, as marine organisms moved to the land and faced a strong selection pressure to conserve an essential micronutrient. It is proposed that these genes no longer fit: the Ancestral-Susceptibility hypothesis posits that hypertension, like other complex modern conditions, is a “disease of civilization” because of a mismatch between ancient genomes and current environment.^17^ According to this theory, ancestral gene variants that promoted efficient sodium retention accrued a selection advantage as humans first developed in the hot and dry African savannah with sodium chloride a scarce nutrient.^18^ The selection pressure changed following the African diaspora, but the latitudinal cline of heat adaptation remained a strong driver of natural selection.^19^ This hypothesis^20^ is broadly supported by the difference in hypertension prevalence in populations: Individuals of white ethnicity have a lower prevalence of salt-sensitive hypertension than do African Americans; populations from hot climates are more susceptible to hypertension than those from cold climates.^19^ At the gene level, there is evidence that ancestral “sodium-conserving” variants contribute to the phenotypic variability of blood pressure. For example, variants in the *AGT* promotor that increase circulating angiotensinogen encoding gene are found at higher frequency in African populations^21^; loss of function variants expected to reduced salt avidity have risen to a higher frequency outside of Africa.^21^ Similar observations have been made for single nucleotide polymorphisms (SNPs) in the genes encoding the α and γ subunits of ENaC^19^ and for kinases regulating major sodium transport proteins.^22^

How should these data be interpreted? One possible inference is that hypertension arises from a mismatch between environment and ancestral salt-conserving variants. Indeed, the gain-of-function variants would impair the pressure natriuresis response, promoting sodium retention. Indeed, such variants in angiotensinogen, for example, are associated with hypertension in the general population.^23^ The persistence of ancestral variants in the molecular machinery for salt conservation becomes deleterious when the environment changes. In our salt-saturated society, a genome aligned with sodium avidity is maladaptive, increasing blood pressure and cardiovascular risk.

However, to contextualize hypertension as a misalignment of ancestrally favorable “blood pressure” variants is too narrow a view. Recent studies suggest that hypertension is a modern bystander effect of selective pressure imposed to conserve other desirable traits. For example, an ancestral variant in the *APOL1* gene, which encodes apolipoprotein-L1, is observed in higher frequency in African Americans, contributing to higher rates of cardiovascular and renal disease.^24^ The disease-causing mechanism is not defined, but it is likely that the positive evolutionary selection pressure on the ancestral variant reflects improved protection against infection by *Trypanosoma brucei,* which causes sleeping sickness, rather than a beneficial effect on blood pressure homeostasis.

A similar picture is emerging for gain-of-function variants in *UMOD*, the gene encoding uromodulin (Tamm-Horsfall) protein. Common gain-of-function SNPs in *UMOD* associate with hypertension, low glomerular filtration rate, and risk of renal disease.^25^, ^26^, ^27^, ^28^ The evolutionary genetics of 1 “risk” SNP (rs4293393) was examined further and identified as the ancestral allele based on expression in the genomes of nonhuman primates.^29^ Further sequencing of ancient hominid genomes identified a protective allele, but this is now found only at low frequency. Overall, this indicates that the evolution of modern man placed a strong selection pressure in favor of the ancestral, risk-associated allele, probably because this confers protection against bacterial urinary tract infection and regulates the innate immune system.^29^

What does Evolutionary Medicine tell us about hypertension? First, these studies underscore the necessity to return to a more “primitive” diet, low in sodium and high in potassium. Second, they can provide new mechanistic insights into blood pressure control: the study of ancestral variants is identifying new loci associated with hypertension, including kinases that regulate ENaC and sodium chloride co-transporter (NCC).^22^

### Salt Appetite and Blood Pressure

Early terrestrial animals evolved highly effective strategies to conserve sodium, and it is evident that both afferent (i.e., salt taste and hunger; gastrointestinal absorption) and efferent (i.e., renal excretion; sweat) arms of salt homeostasis engage a conserved molecular framework of sodium transport proteins and regulatory kinases (Figure 2). Mutations in the genes encoding these key proteins cause Mendelian (monogenic) blood pressure disorders, all of which have an impact on sodium homeostasis.^30^

**Figure 2:**
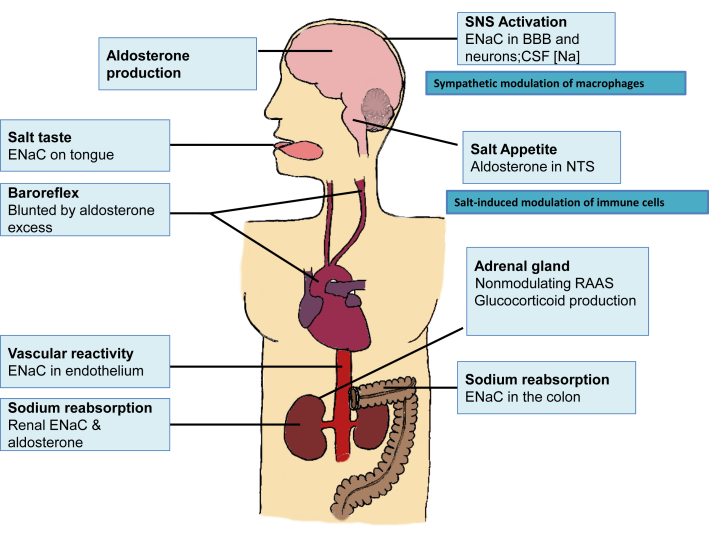
Molecular mechanisms under the control of the renin−angiotensin−aldosterone (RAAS) system are expressed in multiple sites that influence sodium homeostasis and blood pressure. BBB, blood−brain barrier; CSF, cerebrospinal fluid; ENaC, epithelial sodium channel; NTS, nucleus of the solitary tract.

This framework is exemplified in the principal cell of the distal nephron (Figure 3). Aldosterone is a major regulator of sodium balance: activation of the mineralocorticoid receptor stimulates sodium transport through coordinated activation of the Na,K-ATPase in the basolateral membrane and the epithelial sodium channel (ENaC) in the apical membrane. This process is underpinned by activation of serum and glucocorticoid induced kinase 1 (sgk1) to promote ENaC insertion and to suppress ubiquitination and retrieval through Nedd4-2, prolonging ENaC retention in the apical membrane. Additional regulation is achieved by the enzyme 11β-hydroxysteroid-dehydrogenase type 2 (11βHSD2), which converts “active” glucocorticoids into derivatives that do not activate the mineralocorticoid receptor (MR).^31^

**Figure 3:**
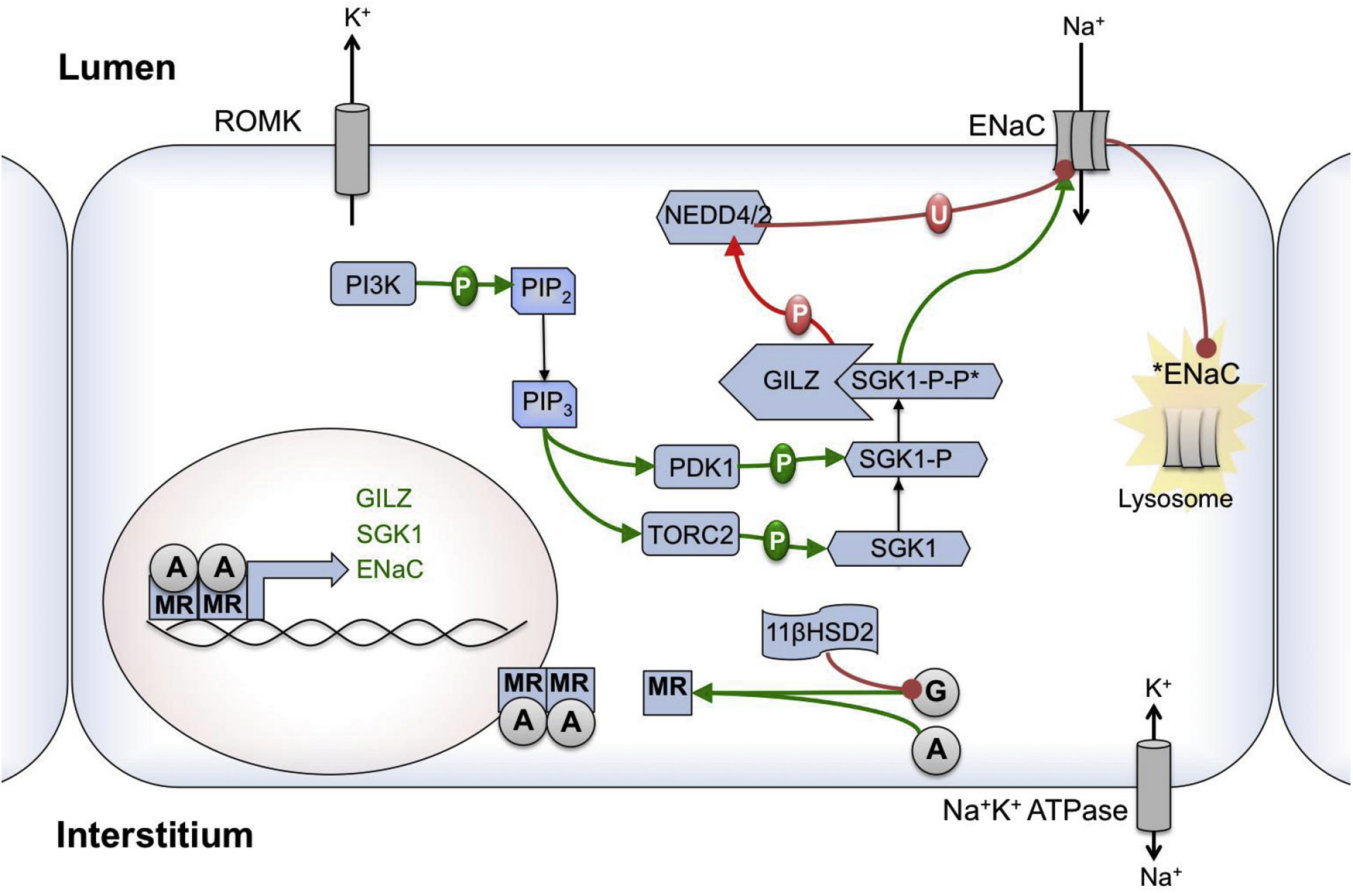
The molecular framework for sodium transport in the principal cell of the collecting duct. Aldosterone is a regulator via the mineralocorticoid receptor, activating transport through a network of regulatory kinases. The enzyme 11β-hydroxysteroid dehydrogenase metabolizes cortisol to cortisone, which does not activate the mineralocorticoid receptor (MR). Glucocorticoids can activate the epithelial sodium channel (ENaC) when in excess.

The same molecular machinery contributes to sodium absorption in the GI tract, activation of salt-taste receptors on the tongue,^32^ control of sodium appetite and sympathetic drive by the brain,^33^, ^34^, ^35^ and also endothelial function/peripheral vascular tone.^36^ Conservation of mechanisms across multiple systems makes evolutionary sense, but how these integrate to control blood pressure is not well defined. In the rest of this article, we focus on mineralocorticoid receptors and the control of salt intake.

Salt appetite is often overlooked as a factor for hypertension and, despite evidence of benefit, compliance with restricted salt intake is poor.^37^ This, of course, reflects the abundance of salt in modern foods, but there may also be a physiological context: heart failure patients show increased preference for salty foods,^38^ and mammals have evolved pathways in the brain that evoke salt appetite in response to sodium/volume depletion.^39^ For example, intracerebrovascular infusion of aldosterone increases blood pressure without altering renal function in rats.^40^ Similarly, intracerebrovascular infusion of 11βHSD2 inhibitors causes hypertension in rats, without any measurable effect on whole-body sodium balance.^41^ 11βHSD2 metabolizes cortisol and protects MR from overactivation by glucocorticoids.^31^ Null mutations in the encoding gene cause the Mendelian syndrome of Apparent Mineralocorticoid Excess. Hypertension in this setting is severe and considered to be renal in origin, as the enzyme is abundantly expressed in the distal nephron.^31^ Nevertheless, patients also have strong salt appetite despite suppressed aldosterone,^42^ and in the general population loss-of-function variants in HSD11B2 positively associate with sodium intake.^43^ MR co-localizes with 11βHSD2 in only a few areas of the brain, populations of neurons that can be considered as classical “aldosterone” target cells.^44^ Several lines of evidence suggest that these 11βHSD2-expressing neurons contribute importantly to salt appetite and blood pressure control. In rats, 11βHSD2-expressing neurons in the nucleus of the solitary tract (*nucleus tractus solitarius*; NTS) are selectively activated by sodium depletion and rapidly inactivated when salt appetite is satiated.^45^ In mice, global genetic deletion of 11βHSD2 causes hypertension and renal sodium retention.^46^ Deleting the enzyme in the NTS alone does not change blood pressure and does not impair renal function.^47^ Nevertheless, these “brain knockout” mice provide strong evidence indicating that that 11βHSD2 activity in the NTS normally exerts a significant influence on sodium homeostasis and BP control by regulating MR activation. Deletion of the enzyme only in the NTS uncovers an innate salt preference such that salt intake increased 3-fold in the absence of any overt physiological driver to consume salt. Furthermore, this increased salt intake induced hypertension: the effect was permissive, as BP did not rise in control mice fed the same high level of salt. This suggests that aldosterone-sensitive neurons in the NTS normally co-ordinate a response to increased salt intake such that blood pressure does not rise. The mechanisms are not yet resolved, but the knockout animals had an exaggerated pressor response to catecholamine and an impaired baroreflex. Other studies show that increased ENaC expression in the choroid plexus and in neurons promotes an exaggerated pressor response to dietary salt.^34^ Important here may be the homeostatic regulation of sodium concentration in the cerebrospinal fluid: small elevations (∼5 mmol/l) increase sympathetic outflow, which causes hypertension by peripheral vasoconstriction and by direct activation of sodium transporters including NCC^48^ and ENaC,^49^ shifting the pressure natriuresis curve to the right and reducing the slope of the response.

It is not certain whether MR activation in the CNS is determined by aldosterone penetrating the blood−brain barrier or whether aldosterone is also produced in the brain.^50^ Recent evidence suggests that central synthesis is promoted by peripheral aldosterone excess to amplify the hypertensive response to salt.^35^

Dysregulation of central salt-regulating pathways can compromise long-term adherence to restricted sodium intake and promote hypertension. Can these processes be targeted to improve cardiovascular outcome? Certainly the brain and the kidney “talk” to each other, and bilateral renal denervation has gained considerable traction as a device-based approach to manage blood pressure in patients with resistant hypertension.^51^, ^52^ Interest has waned considerably since the first randomized, double-blinded, placebo-controlled trial (Symplicity HNT-3) failed to reach its primary endpoint and was halted early in 2014.^53^ Experimental hypertension, a setting in which effective denervation can be rigorously determined, shows that interrupting brain−kidney communication is more effective in some models (e.g., obesity) than others (e.g., Dahl salt-sensitive). *Post hoc* analysis of Symplicity-HTN3 found benefit of denervation in hypertensive patients with obstructive sleep apnea,^54^ suggesting that patient stratification might lead to a renaissance of this therapeutic approach.

### Summary

For most of mankind’s existence, sodium chloride was a scarce nutrient. This scarcity gave great economic value to salt and shaped the formation and customs of our societies, both ancient and modern. It is also reflected in our DNA, encoding the multiple interlocking pathways that efficiently control salt balance. However, our salt intake is now habitually high, and these genes no longer fit: blood pressure rises, and cardiovascular disease is the leading cause of global mortality. It is clear that BP homeostasis is intimately associated with sodium homeostasis and the distribution of sodium between fluid compartments and within tissues. Research has given us a more sophisticated understanding of blood pressure control, revealing a dynamic interplay among hormonal, neuronal, and immune systems. Our habitually high salt intake promotes abnormal interactions and causes hypertension. This improved understanding may help us to develop therapeutic and lifestyle interventions to tame the “silent killer.”

## Disclosure

All the authors declared no conflict of interest.
